# Novel psychotherapies for insomnia

**DOI:** 10.1111/jsr.14470

**Published:** 2025-01-31

**Authors:** Marie Angelillo, Jaap Lancee, Elisabeth Hertenstein

**Affiliations:** ^1^ Department of Psychiatry, Faculty of Medicine University of Geneva Geneva Switzerland; ^2^ Department of Clinical Psychology University of Amsterdam Amsterdam the Netherlands

**Keywords:** acceptance and commitment therapy, cognitive behavioural treatment, insomnia, mechanisms, non‐response

## Abstract

Insomnia disorder, characterized by a complaint of reduced sleep quality or quantity and associated daytime impairment, is highly prevalent and associated with reduced quality of life and productivity. Cognitive behavioural therapy for insomnia (CBT‐I) is the current first‐line treatment for chronic insomnia disorder. Here, we outline our perspective for the future optimization of psychotherapeutic treatment for insomnia. We identified the following areas as the most promising: first, optimizing efficacy of the CBT‐I protocol; second, developing diagnostic and therapeutic approaches for non‐responders and partial responders; and third, advancing widespread implementation of psychotherapy for insomnia. More specifically, we outline how the current CBT‐I protocol could be optimized through an improved understanding of treatment mechanisms, and discuss the potential of adaptive treatment strategies. Another promising approach for improving the current CBT‐I protocol is using add‐ons such as physical exercise or circadian‐based interventions. Both may be promising in certain subgroups of patients with insomnia. In terms of non‐response, we identify acceptance and commitment therapy for insomnia (ACT‐I) as a promising treatment for non‐responders to CBT‐I. ACT‐I, however, still needs to be evaluated in actual non‐responders to CBT‐I. Implementing CBT‐I in clinical practice is still one of the major challenges at hand. We outline how brief treatment, targeted treatment for challenging patient groups, and digital treatment may help improve implementation. For a future research agenda, we suggest that further research into treatment mechanisms, randomized–controlled trials in non‐responders to CBT‐I, and a focus on implementation science have a potential to bring the field forward.

## 1 INTRODUCTION

According to current guidelines, cognitive behavioural therapy for insomnia (CBT‐I) is the treatment of choice for patients with chronic insomnia (Riemann et al., 2023). The two main pillars of CBT‐I are the behavioural and cognitive components. The behavioural components contain bedtime restriction (also known as sleep restriction) and stimulus control. In the cognitive components, the focus is on selective attention, a potential discrepancy between subjective sleep perception and objective measurements, and dysfunctional beliefs about sleep (Jansson‐Frojmark & Norell‐Clarke, 2018). CBT‐I as a treatment package including the modification of sleep‐related behaviour and sleep‐related thoughts, education and relaxation has been known since the 1990s. Results of a randomized clinical trial suggest that four individual, biweekly sessions represent the optimal dosage of CBT‐I (in contrast to one, two or eight sessions; Edinger et al., 2007).

The efficacy of CBT‐I has since been proven in numerous randomized–controlled trials (RCTs) and meta‐analyses (Edinger et al., 2021; van Straten et al., 2018). One may argue that we have reached a satisfactory status that leaves little room for improvement. However, the efficacy of CBT‐I is good, but not excellent. While effect sizes are generally large in clinical trials, partial response is rather the norm than the exception. For example, while most patients show a clinical response to CBT‐I, about 50% of the patients who receive CBT‐I do not fully remit (Edinger et al., 2021). It is, therefore, crucial to deepen our understanding of the treatment mechanisms of CBT‐I to identify and optimize its core therapeutic components.

Even with such an enhanced and well‐tuned CBT‐I protocol, it is realistic that there still will be a group of non‐responders. For those non‐responders, current evidence‐based guidelines recommend medication that is often associated with the risk of side‐effects, tolerance, loss of effect and dependency (Riemann et al., 2023). Moreover, medication is only indicated for short‐term use, at least for the medications sufficiently investigated so far. Therefore, a second‐line psychotherapeutic approach based on another treatment mechanism than CBT‐I would be highly desirable.

The wide‐scale implementation of CBT‐I remains one of the major challenges for clinical sleep research of our time. It was estimated that only 10% of general practitioners recommend CBT‐I, and even less patients with insomnia actually receive it (Mulder et al., 2023). Implementation in demanding treatment settings such as psychiatric hospitals is a particular challenge. An improved understanding of treatment response, in different patient groups and for different parts of CBT‐I, could help optimize treatment, for example, in the form of adaptable treatment modules.

In this article, we argue that innovation is still much needed to improve treatment for insomnia. We think the following areas are most promising: (1) optimizing efficacy of the current CBT‐I protocol; (2) developing diagnostic and therapeutic approaches for those who do not respond to CBT‐I; (3) advance widespread implementation. The aim of this article is to summarize the current state of research and identify promising areas for future research in these three areas.

## 2 OPTIMIZING **CBT** ‐I

Treatment development and a promising research agenda for treatment optimization need to be based on a solid, evidence‐based conceptualization of the disorder at hand. The current conceptualization of insomnia this article is based on is a conceptualization of insomnia as a “predominant complaint of dissatisfaction with sleep quantity or quality”, as defined in the DSM‐5 (American Psychiatric Association diagnostic and statistical manual of mental disorders (DSM*‐5®*), 2013), with disorder‐specific cognitions, emotions and behaviours maintaining the problem. In this conceptualization, patients with insomnia generally have the capacity to build up sleep pressure and maintain a stable circadian rhythm. Under this assumption, a focus on cognitive and behavioural approaches as outlined in this article makes sense. Cognitive and behavioural interventions have the capacity to re‐adjust sleep pressure, arousal levels and circadian rhythms. Therefore, while targeting mainly subjective sleep, they may (or may not) have secondary effects on objective sleep. It is well known that changes in subjective sleep and objective sleep often do not go hand in hand. In the literature, the distinction between interventions targeting subjective versus objective sleep is often not clear enough, with a potential for confusion on the side of patients and healthcare providers. Future research needs to ensure that proposed interventions are based on a solid evidence‐based concept of insomnia that aligns with the rationale and approach of the investigated intervention. In the following section, we will first discuss treatment mechanisms and adaptive treatment strategies, followed by potential add‐ons to CBT‐I such as exercise and circadian‐based interventions.

### 2.1 Understanding treatment mechanisms and the potential of adaptive treatment strategies

The aim of research into treatment mechanisms is to differentiate between highly effective treatment components and less effective treatment components—potentially allowing for more refined, efficient treatment packages. In addition, research into symptom‐specific effects has the potential to pave the way into tailored, precise treatments for patients with specific symptom constellations. This research promises both more effective and more efficient treatment packages.

In terms of components of CBT‐I, there is solid evidence underpinning the efficacy of bedtime restriction therapy as a stand‐alone treatment (Maurer et al., 2021). Behavioural components of CBT‐I (bedtime restriction and stimulus control) are generally considered the most effective parts of CBT‐I (Steinmetz et al., 2024). The authors of a three‐armed RCT directly comparing full CBT‐I, behavioural therapy and cognitive therapy concluded that full CBT‐I had the best treatment effects, whereas effects of behavioural therapy were faster but less long‐lasting, and effects of cognitive therapy alone were slower and more sustained (Harvey et al., 2014). A recent meta‐analysis underlines that the critical elements to achieve remission of insomnia are restriction of time in bed, stimulus control and cognitive restructuring (Furukawa et al., 2024).

Network intervention analysis is a newly developed statistical approach that allows for a fine‐grained analysis of symptom‐specific effects of different treatments (Blanken et al., 2019). A recent network intervention analysis of cognitive therapy and behavioural therapy concluded that the effects of the two interventions are consistent with their respective theoretical underpinnings, for example, cognitive therapy had effects on worry, dysfunctional beliefs and threat monitoring, whereas behavioural therapy had effects on sleep efficiency, sleep maintenance and maladaptive sleep‐related behaviours (Lancee et al., 2022). In another study, it was demonstrated that while behaviour therapy and zolpidem had similar effects on insomnia severity at post‐test, behaviour therapy showed larger effects on subjective night‐time wakefulness and zolpidem showed larger effects on daytime functioning (Lancee et al., 2023).

A potential conclusion from behavioural treatment being an effective stand‐alone may be to offer only behavioural treatment (bedtime restriction and stimulus control) as first‐line treatment. A couple of studies on stand‐alone bedtime restriction and stand‐alone stimulus control have been published, with promising effects (Jansson‐Frojmark et al., 2023; Maurer et al., 2021). Recently, a large‐scale study in the UK has demonstrated the efficacy of stand‐alone bedtime restriction therapy provided by healthcare nurses (Kyle et al., 2023). Our research group has also demonstrated large treatment effects of bedtime restriction alone in a telephone‐guided setting (Looman et al., n.d.). A potentially promising strategy would be to offer an adequate dose of behavioural treatment to every patient with insomnia, as a first‐line treatment. This behavioural approach may be sufficient for some of the patients and others may need more. The group needing more may then progress to more extensive treatment (e.g. CBT‐I or acceptance and commitment therapy for insomnia [ACT‐I]). This would thus imply a first lower‐intensity step in the stepped‐care model. Clearly, this suggestion in finetuning the first‐line treatment format and altering how people best progress in a stepped‐care model first needs to be empirically tested. For this reason, we need trials comparing behavioural therapy with CBT protocols, but also trials investigating the adaptive treatment format, monitoring, allocation and the sequence of treatments. Until these studies are carried out, we fully underline the recommendation for CBT‐I as first‐line treatment.

Another important issue is to find out how people should progress to new treatment steps. Here an adaptive treatment protocol may be interesting. In adaptive treatment formats, the goal is to optimize resource distribution and reduce patient dropouts. A potentially promising strategy is to adapt treatment in an early treatment phase, such that all patients start with low‐intensity treatment while their progress is monitored. If treatment progress is insufficient in the early stages of treatment (e.g. after 2 weeks), treatment is directly adapted to their needs. Recently, an adaptive treatment procedure was successfully tested by first offering digital CBT‐I (dCBT‐I), and adjusting the amount or type of support in the next stage (Forsell et al., 2019). To this end, the authors developed a semiautomated algorithm assessing risk of treatment failure early in the treatment process, and an adapted treatment strategy that increased the chance of treatment success in at‐risk patients.

### 2.2 CBT‐I with add‐ons

Neurobiological, technical and other add‐ons have the potential to increase the efficacy of CBT‐I. Here, we briefly summarize the evidence for physical exercise, circadian‐based treatment strategies and targeted memory activation as potentially promising add‐ons to CBT‐I. We also comment on wearable sleep trackers (WSTs) and non‐invasive brain stimulation (NIBS) techniques that have been proposed in the literature but come with potential pitfalls and limitations.

#### 2.2.1 Physical exercise

Exercise interventions for insomnia involve structured physical activities that aim to improve insomnia symptoms. A recent meta‐analysis of 19 primary studies found significant improvements of both subjective and objective sleep with exercise in a mixed sample of patients with diagnosed insomnia and people with insomnia complaints without diagnosis (Riedel et al., 2024). However, exercise as an insomnia treatment has not yet been systematically compared with CBT‐I. From a clinical point of view, a combination of both seems most promising and should be investigated in future RCTs.

Most exercise‐based interventions follow WHO guidelines of 150 min of moderate to vigorous physical activity per week (Passos et al., 2023; Riedel et al., 2024). Among different types of exercise, aerobic exercise—especially at higher intensity—shows the strongest results in improving sleep. Stretching and resistance training are less effective, but still show better results than control groups. Low‐intensity, unstructured exercise (e.g. general stretching) has limited benefits. Furthermore, sleep outcomes vary significantly based on exercise intensity, age, gender and other comorbidities (Passos et al., 2023; Riedel et al., 2024). Additionally, the timing of exercise plays a crucial role. Morning or afternoon physical activity tends to improve sleep, while exercising late at night can negatively impact insomnia symptoms (Passos et al., 2023).

Although exercise on its own improves sleep, it is mainly used and recommended as an adjunct to treatments like CBT‐I. Combining exercise with CBT‐I is considered more effective, as it addresses multiple facets of insomnia, such as sleep drive, circadian rhythm, hyperarousal and behavioural factors. This combination can be particularly relevant in cases where CBT‐I alone is not sufficient.

Key research questions include how to optimize exercise interventions by exploring the most effective type, intensity, timing and frequency for different populations. Especially, timing questions are of high interest for insomnia treatment: exercise early in the morning, especially when done outside, might help advance the circadian rhythm and therefore address the frequent problem of a delayed rhythm. Exercise too late in the evening, shortly before bedtime, is often said to be detrimental for sleep quality. Both questions should be addressed systematically in future research. Because patients typically have more time at their disposal due to bedtime restriction, they may be encouraged to make use of this time in terms of regular exercise. Finally, the role of technology, such as apps and wearables, in promoting exercise adherence among individuals with insomnia should be further investigated (Passos et al., 2023; Riedel et al., 2024).

#### 2.2.2 Circadian‐based add‐ons

Circadian‐based interventions for insomnia are treatments that aim to regulate or realign the circadian rhythm in order to alleviate insomnia symptoms. Whereas restriction of time in bed addresses sleep pressure or “process S” in the two‐process model of sleep regulation, circadian‐based add‐ons more directly address “process C” (Borbély et al., 2016). Common approaches include bright light therapy or strategic use of dim light and blue‐blocking glasses, as well as melatonin supplementation. Other factors that can influence process C, such as temperature regulation, meal timing, physical activity and social cues, may also be incorporated. The core idea remains the same: to provide process C signals to the body's metabolism, promoting sleep at the desired times (Borbély, 1982). Most research on circadian‐based interventions focuses on light therapy. In a study comparing CBT‐I, circadian rhythm support and their combination in patients with insomnia at high risk for depression, circadian rhythm support did initially not boost efficacy of CBT‐I but improved maintenance of beneficial effects at follow‐up (Leerssen et al., 2022).

The findings on light therapy are mixed, highlighting the importance of correctly timing these interventions (Ell et al., 2023; Swanson & Raglan, 2023). High variation can also be explained by the fact that circadian add‐ons are only relevant for a subgroup, namely those patients who have a shifted circadian phase—mostly sleep‐onset insomnia due to a phase delay (Lack et al., 2023). Light therapy can be timed in three ways: morning light exposure and/or evening dim light exposure (e.g. red dim light, blue‐blocking glasses), which advances sleep–wake rhythms and helps people fall asleep earlier, or evening light exposure, which causes a phase delay, helping individuals stay asleep longer. Chambe et al. (2023) report promising results from light therapy, particularly for improving sleep maintenance (wake after sleep onset), but little impact on sleep latency, total sleep time or sleep efficiency. The overall effects are moderate, with light intensity emerging as a key factor—higher intensity in morning light exposure tends to yield stronger effects.

Some findings indicate that chrono‐nutrition patterns—such as late final meals and more frequent, irregular eating—are associated with poor sleep quality and mental health outcomes (Nunes et al., 2024; Yan et al., 2024). Alternatively, other studies report that meal timing has no significant effect on subjective sleep problems (Cienfuegos et al., 2022; van Egmond et al., 2020). Regular lifestyle patterns are associated with better sleep, while unstable social rhythms are linked to poorer sleep quality (Meng et al., 2023; Monk et al., 2003). These findings are correlational; therefore, it is unclear whether increasing regularity alone would improve insomnia or if other factors are at work (e.g. unemployment, shift work, etc.).

Overall, the best evidence base exists for light therapy, while other circadian‐based interventions are under‐investigated. It seems interesting and generally promising to combine circadian rhythm support and behavioural treatment for insomnia. Future research should focus on the identification of subgroups for whom circadian‐based interventions are promising—for example, those with a shifted rhythm in the sense of a phase‐delay or a phase‐advance.

#### 2.2.3 Targeted memory reactivation (TMR)

In TMR, memory content is paired with a cue (e.g. an odour or a tone) during learning. Using this cue, the memory is reactivated during sleep, with beneficial effects on learning performance. Such techniques have been used successfully to enhance the efficacy of imagery rehearsal therapy for nightmares (Schwartz et al., 2022). Here, the memory of a more positive storyline of a nightmare was paired with a sound, and reactivated with the same sound during rapid eye movement (REM) sleep. Such techniques may be suitable to enhance CBT‐I, for example, with the aim of increasing memories of relaxation during sleep (similar to the approach tested in Borghese et al., 2022). This, however, is still in a very early stage, and no studies of the use of TMR in insomnia therapy have been published yet. TMR usually requires online electroencephalogram (EEG) monitoring to allow for a targeted application of the cue during a specific sleep stage. Wearables for at‐home use may allow for easy implementation in the near future. In principle, online EEG monitoring could also be used to identify wake periods, and send reassuring, calming stimuli with the aim of making it easier for patients to fall back asleep.

#### 2.2.4 Wearable sleep trackers (WSTs)

The WSTs are designed to record and monitor sleep‐related data. They could in some cases be valuable add‐ons to CBT‐I; however, they come with several problems and potential pitfalls. Therefore in general, in our view, sleep tracking based on subjective perception, with the help of sleep diaries, should remain the first choice for monitoring treatment success and for adapting the sleep window. The designs of WSTs range from smartwatches to more specialized devices like finger‐worn sensors or wristbands (Chiang & Khosla, 2023; Haghayegh et al., 2019). These devices primarily aim to support behaviour changes related to health, by providing consumers access to their health data including sleep and activity patterns, and by offering feedback and health recommendations (Chiang & Khosla, 2023).

Subjective sleep is at the core of insomnia complaints, diagnosis and CBT‐I treatment (Riemann et al., 2023). WSTs, in contrast, aim to provide objective sleep‐related data. The use of WSTs therefore comes with a conceptual problem: the potential confusion of subjective and objective sleep. Therapeutic approaches over‐emphasizing the role of objective sleep may be misleading and counterproductive for patients and clinicians. Replacing patients' sleep diary monitoring with “objective” data from WSTs therefore seems generally problematic.

The WSTs may, however, be helpful when it comes to monitoring bed‐ and rise‐times. Here, they can provide patients and clinicians with valuable information about treatment adherence, such as comparing objective time in bed with prescribed time in bed, detecting whether a patient has followed stimulus control recommendations, or send reminders that the prescribed bedtime is approaching. Furthermore, these devices can help track and display adherence progress over time, encouraging adherence to treatment (Lai et al., 2023). Additionally, WST data may be used to address differences between subjective sleep perception and objective measurement. This, however, must be applied with caution, due to inter‐ and intra‐individual differences and potential error in measurement (Scott, Lechat, et al., 2023).

Early evidence suggests that patients who completed online CBT‐I, with or without a WST, achieved similar treatment outcomes (Luik et al., 2018). However, for some individuals, WSTs may be counterproductive. Over‐focus on these devices, known as “orthosomnia”, can interfere with treatment by causing patients to fixate on their sleep data (Baron et al., 2017). This suggests that WSTs may be more suitable for certain subgroups of patients with insomnia than others.

Finally, as technology advances, WSTs are likely to improve significantly. New sensors, machine learning and AI developments hold the potential to enhance how these devices process data and expand their clinical applications (Chiang & Khosla, 2023).

#### 2.2.5 Intensive sleep retraining

Another potential application for WSTs is intensive sleep retraining. It is a relatively new behavioural treatment approach for patients with insomnia that involves monitoring of objective sleep and waking patients consistently as soon as they fall asleep. This has two aims: to build sleep pressure through sleep deprivation; and to make patients aware that they had just fallen asleep. Previously, this treatment was only available in costly lab settings but, with WSTs, it can now be done at home with tailored adjustments, such as modifying the sleep deprivation period based on patient needs (Bensen‐Boakes et al., 2023; Lack et al., 2019; Scott, Lechat, et al., 2023). This approach should be investigated in future research.

#### 2.2.6 Non‐invasive brain stimulation (NIBS)

The NIBS techniques are used to modulate brain activity without the need for surgical procedures, primarily in the treatment of neurological and psychiatric disorders. The most common methods include transcranial magnetic stimulation and transcranial electric stimulation. Additionally, transcutaneous auricular vagus nerve stimulation, transcutaneous vestibular nerve stimulation, forehead cooling, and auditory stimulation have also been explored as potential treatments for insomnia (Krone et al., 2023).

Significant limitations and uncertainties remain regarding the potential of NIBS for treating insomnia, not only due to methodological issues in many studies (Krone et al., 2023), but also because the treatment rationale and goal is rather questionable. NIBS was initially thought to hold potential for improving objective sleep measures—as an add‐on to CBT‐I that aims mainly at improving subjective sleep. Fundamental improvements of insomnia are, however, unlikely with this approach as following a contemporary conceptualization of insomnia, sleep–wake regulatory systems in the brain are largely intact in patients with insomnia, the disorder is defined primarily as a disruption of subjective sleep, with only minor alterations of objective sleep that may be caused by psychological arousal and anxiety rather than altered brain function. While some still see promise in NIBS for insomnia, consistent results have yet to be demonstrated.

Another approach has been to shift the treatment target of NIBS from sleep parameters to daytime functioning, an integral aspect of insomnia disorder. The rationale is that cognitive workload and daytime activity may increase sleep pressure, suggesting that enhancing daytime function could indirectly improve sleep. Brain stimulation techniques have been explored for this purpose, though their effectiveness specifically in patients with insomnia has yet to be demonstrated (Krone et al., 2023).

To determine whether NIBS could be effective for treating insomnia, further research is essential to clarify the disorder's pathophysiology and underlying mechanisms. The role of NIBS will depend on strong evidence linking the core pathology of insomnia to brain function. However, if insomnia is found to stem primarily from anxiety, misperception and subjective experience, then NIBS may have limited relevance for its treatment. If anything, NIBS may be most promising to improve daytime function including cognitive performance.

## 3 DEALING WITH NON‐RESPONSE

Response and remission in insomnia research are typically defined in terms of changes in the summary score of the Insomnia Severity Index from baseline to after treatment. A change score of 8 or more points is considered as a clinically relevant response (Morin et al., 2011). Remission is defined as a score below 8 after treatment (Morin et al., 2009). Good adherence to behavioural recommendations and a higher severity of insomnia at baseline have been identified as predictors of response (Edinger et al., 2023; Scott, Cheung, et al., 2023). In fact, what is at first glance non‐response may in some cases actually be non‐adherence. Interventions aiming at improving adherence to behavioural and cognitive interventions, such as regular ongoing support and a possibility to discuss open questions and challenges directly with a therapist, may have high potential to improve both adherence and response. In a recent large clinical trial, 36 of 88 treatment completers were remitted after behavioural therapy for insomnia (41%; Morin et al., 2020). Response rates vary between trials, with 52% responders at post‐treatment in a recent trial (El Rafihi‐Ferreira et al., 2024), and 60% in an older trial (Morin et al., 2009), and typically increasing response rates at later follow‐up measures.

New treatments, such as ACT‐I, have been suggested as a second‐line treatment for non‐responders. However, at this point in time, it remains largely unknown whether ACT‐I is suitable as a second‐line treatment for non‐responders, because existing trials have not been conducted in non‐responders. In the following paragraphs, we will discuss ACT‐I and mindfulness‐based therapy as potential second‐line treatments.

### 3.1 Acceptance and commitment therapy (ACT)

Increased attentional focus on sleep‐related cues, sleep‐related worry and sleep effort can result in a self‐perpetuating vicious circle maintaining insomnia (Espie et al., 2006): The more we want to sleep, the less we can. In this scenario, the anxiety towards bad sleep paradoxically becomes one of the main reasons for bad sleep. ACT‐I aims at interrupting this vicious circle by increasing psychological flexibility. The idea is that “letting go of the struggle” will help to improve sleep and quality of life. The second main aim of ACT‐I is to encourage value‐based and goal‐driven behaviour.

In 2022, a systematic review on the efficacy of ACT‐I was published (Paulos‐Guarnieri et al., 2022). In this paper, 11 studies were identified, of which six were RCTs. Almost all papers reported promising effects of ACT‐I on clinically relevant outcomes, but the RCTs were conducted in relatively small sample sizes with methodological limitations. Since the publication of the systematic review, two more clinical trials with a larger sample size have been published (El Rafihi‐Ferreira et al., 2024; Hertenstein et al., 2024). Hertenstein et al. combined ACT with bedtime restriction in a six‐session group treatment. This treatment had large effects on the reduction of insomnia severity and the improvement of sleep‐related quality of life. Effects were stable at a 6‐month follow‐up. There was no significant difference in the efficacy of ACT plus bedtime restriction and CBT‐I. El Rafihi‐Ferreira et al. found that both ACT‐I and CBT‐I were superior to waitlist, but CBT‐I was superior to ACT‐I for the improvement of insomnia severity in this study (El Rafihi‐Ferreira et al., 2024). Of note, whereas Hertenstein et al. investigated ACT combined with bedtime restriction, El‐Rafihi Ferreira et al. investigated ACT‐I only, without elements of CBT‐I. For the reduction of insomnia severity, CBT‐I was superior to ACT‐I alone at post‐treatment and follow‐up. Response and remission rates were higher in CBT‐I than ACT at post‐treatment, but not significantly different at follow‐up. In conclusion, CBT‐I is superior to ACT‐I alone, whereas ACT combined with bedtime restriction seems to be equally effective as the first‐line treatment. Non‐inferiority has not yet been formally investigated.

A potential problem, however, is that if bedtime restriction is included in the ACT‐I protocol, it could be that the same people that are not responding to CBT‐I may also be non‐responders to the ACT protocol combined with bedtime restriction. ACT‐I as a stand‐alone protocol may not be as effective but may tailor to a specific subset of people not accepting or not responding to bedtime restriction. This is, however, still an open question that has not yet been investigated in actual non‐responders.

Of the CBT alternatives that may be suitable as a second‐line treatment for non‐responders to CBT‐I, ACT‐I seems very promising. Therefore, studies investigating ACT (with and without bedtime restriction) for actual non‐responders to CBT‐I, instead of previously untreated patients, are now needed. In a small pilot study, Hertenstein and colleagues showed that treatment effects with non‐responders are promising especially for quality of life improvements; however, methodological shortcomings of this uncontrolled study limit generalizability (Hertenstein et al., 2014). A future research agenda should also include studies shedding light on treatment mechanisms, and should clarify how treatment needs to be tailored to maximize efficacy for different patient groups. Work in this area is carried out in an ongoing study on stand‐alone ACT‐I (Looman et al., 2024). In our view, this line of research should be one of the priorities in the field of insomnia treatment.

### 3.2 Mindfulness‐based therapy

Mindfulness is part of ACT, but can also be offered as a stand‐alone or combined with other treatments. The programs using mindfulness to treat insomnia generally aim to reduce sleep‐related hyper‐arousal via mindfulness principles (e.g. fostering non‐judgemental awareness of the present moment) and meditation practices (Ong et al., 2012). The most common one is mindfulness‐based therapy for insomnia (MBTI), which combines meditation practices with behavioural strategies (i.e. bedtime restriction, stimulus control and sleep education). The structure is typically eight weekly group sessions, with daily 30–45‐min home guided meditation. The main difference with CBT‐I is the cognitive component, where mindfulness focuses on the metacognitive awareness processes to reduce arousal versus challenging dysfunctional beliefs in CBT‐I. As outlined for ACT‐I, combining mindfulness with bedtime restriction is promising; however, at the same time the inclusion of bedtime restriction may also mean that it is not the best option for people who did not respond well to CBT‐I because they struggled with bedtime restriction.

Other mindfulness‐based programs exist, with potential impact on insomnia, like mindfulness‐based stress reduction, mindfulness‐based cognitive therapy, which usually targets depression, or, more specifically related to insomnia, mindfulness‐relaxation therapy for insomnia (Ong et al., 2014; Ong & Kalmbach, 2023; Perini et al., 2023). What all these programs have in common is their focus on acceptance and mindful awareness.

The MBT‐I (mindfulness plus bedtime restriction) seems to be the most promising mindfulness‐based program for insomnia, showing reductions in insomnia severity that are maintained at follow‐up (Perini et al., 2023).

The MBT‐I seems to have benefits on emotion regulation (Ong et al., 2012; Ong et al., 2018) and tends to have high levels of patient engagement (Ong & Kalmbach, 2023). Although non‐inferiority between MBT‐I and CBT‐I has been demonstrated, as only a few studies have directly compared mindfulness with CBT‐I, CBT‐I continues to be considered the gold‐standard for treating primary insomnia (Ong & Kalmbach, 2023).

Open questions for future research include exploring the characteristics of patient populations (e.g. which patients are likely to benefit from MBT‐I and what predicts treatment response), the context of insomnia (e.g. whether MBT‐I is more suited for primary or comorbid insomnia, and whether it functions best as an adjunct or stand‐alone treatment), the mechanisms of action (e.g. breaking down MBT‐I components to target specific mindfulness practices), treatment integration (e.g. how MBT‐I interacts with other interventions), treatment delivery (e.g. remote or digital formats), and long‐term sustainability (e.g. follow‐up strategies and cost‐effectiveness; Ong & Kalmbach, 2023).

## 4 IMPLEMENTATION

Despite its well‐known efficacy, CBT‐I not being implemented in clinical practice is one of the biggest challenges of our time. Implementation science is an emerging research field dealing with the identification of scientific methods and practical strategies that help implement treatments with known efficacy, and de‐implement treatments frequently used but known to be of limited value. Implementation science studies typically consist of different steps, including but not limited to an analysis of the context, the involvement of important stakeholders, theories and strategies for behavioural change. An extensive coverage of insomnia and CBT‐I from the point of view of implementation science would be beyond the scope of this article. However, because we feel that this topic is of particular interest and importance in the field, we will summarize several approaches that have potential to help overcome the implementation crisis. These approaches are tailored programs for implementation in especially challenging settings, brief treatment and digital treatment that may be easier to conduct and therefore easier to implement. In addition, because a recent trial demonstrated the efficacy of stand‐alone bedtime restriction delivered by nurses, broadening the scope of healthcare professionals who provide insomnia treatment may also be a way to improve implementation (Kyle et al., 2023).

### 4.1 Tailored programs for inpatients

Several research groups have developed tailored CBT‐I‐based programs for insomnia in those with more severe psychiatric comorbidity, especially in the inpatient setting (Schneider et al., 2020; Sheaves et al., 2018). A common idea of different programs is to conceptualize a transdiagnostic approach for a certain setting such as inpatient psychiatry, in contrast to specialized programs for each comorbid disorder.

Tailored CBT‐I programs for inpatients with insomnia and co‐occurring psychiatric disorders (Schneider et al., 2020; Sheaves et al., 2018) can be integrated into a multimodal psychiatric treatment approach, such as a modular transdiagnostic model. This approach adjusts treatments to address overlapping symptoms of insomnia and other mental health conditions (Callaway et al., 2023; Sheaves et al., 2018). The focus is on assuring sufficient sleep pressure upon bedtime, circadian rhythm stabilization and empowering inpatients with psychiatric problems to self‐manage their sleep, with guidance from healthcare providers. Designed to be straightforward, flexible and easy to understand, these programs offer a practical approach to treating insomnia in psychiatric inpatients settings (Callaway et al., 2023; Schneider et al., 2020).

Several programs have shown promising results, with feasibility in challenging settings and improvements in both sleep and overall mental health (Sheaves et al., 2018). Key components that have been identified as effective include: bedtime restriction (Callaway et al., 2023; Schneider et al., 2020), circadian adaptation (Callaway et al., 2023; Sheaves et al., 2018), as well as sleep and circadian education (Callaway et al., 2023; Schneider et al., 2020).

Overreliance on sleep hygiene alone, on the other hand, has not been enough to bring about significant improvements (Callaway et al., 2023; Schneider et al., 2020). Additionally, while some programs, like those described by Sheaves et al. (2018), found sleep windows too rigid, others like Callaway and Schneider successfully implemented them (Callaway et al., 2023; Schneider et al., 2020). This suggests that the adaptation of certain CBT‐I modules may need further refinement depending on the context (Schneider et al., 2020).

These tailored programs are designed as alternatives to full CBT‐I and can be effective on their own (Callaway et al., 2023; Schneider et al., 2020). For example, Sheaves et al. (2018) added light therapy to their program, enhancing its effectiveness.

Future research needs to clarify which components are most effective within this modular approach (Callaway et al., 2023), and determine the optimal intensity, target population, setting and duration of treatment (Schneider et al., 2020). There are also open questions about how these programs can be integrated with or without pharmacotherapy, as well as how to ensure long‐term efficacy and the best strategies for follow‐up support after discharge (Sheaves et al., 2018).

### 4.2 Digital treatment

Digitally delivered or internet‐based CBT‐I (dCBT‐I or iCBT‐I) refers to CBT‐I provided through digital platforms (Espie & Henry, 2023). Delivery methods for dCBT‐I vary, including web‐based programs that may be guided (with human support) or non‐guided (fully automated), self‐guided mobile applications and, in some cases, phone‐based CBT‐I with therapists. These digital programs are typically modelled on traditional CBT‐I, incorporating its core components (behavioural, cognitive and educational), though the format may vary. Face‐to‐face CBT‐I remains the gold‐standard; however, its limited availability does not meet the demand. In this context, dCBT‐I can be a valuable alternative for those who might otherwise struggle to access care. The possibilities and efficacy of dCBT‐I have been discussed in several excellent papers (Espie et al., 2019; Hasan et al., 2022), therefore in this article we only briefly mention the important challenges related to optimizing CBT‐I.

One of the main challenges, especially regarding fully automated formats, is that they are often associated with high dropout rates, sometimes reaching close to 50% (Luik et al., 2019). Adverse effects, particularly emerging from bedtime restriction, may contribute to this, but further research is needed to better understand reasons behind dropouts. Hybrid models combining automation with human support might help to reduce dropout rates (Espie et al., 2019). Future studies should compare different delivery methods, investigating the role of human support in improving outcomes. Regarding the latter, we need to investigate which kind of human support patients need and by whom, for example, do we need psychotherapists to provide this support, or would a nurse or lay person be equally effective? For fully automated CBT‐I, a key challenge is finding ways to enhance user engagement and reduce dropouts.

### 4.3 Brief treatment programs

Brief behavioural treatments for insomnia (BBT‐I) are condensed versions of CBT‐I, typically delivered in four sessions over 4–5 weeks. BBT‐I focuses on key behavioural components—stimulus control and bedtime restriction—with the first session covering sleep education and establishing a sleep window. The following sessions involve reviewing sleep diaries, adjusting the sleep window, and discussing adherence. BBT‐I is designed to be accessible, requiring minimal training, and adaptable to a variety of settings, including telehealth, digital platforms and in‐person formats (Bramoweth et al., 2020; Gunn et al., 2019). BBT‐I shows promising results, particularly for its applicability to diverse populations and settings. Studies have shown efficacy, with improvements lasting up to 6 months post‐intervention. The rationale why BBT‐I is assumed to improve implementation is that minimal training is required for providers, making BBT‐I a practical option for general care settings where access to full CBT‐I is limited (Bramoweth et al., 2020; Gunn et al., 2019).

However, challenges remain. BBT‐I is less effective for certain groups, such as older adults with nocturia or people with short sleep duration at baseline (Troxel et al., 2013; Tyagi et al., 2014). Responses also vary among patients with high anxiety or depression (Troxel et al., 2013). More research is needed to assess its effectiveness across broader populations and explore its generalizability, particularly in relation to comorbidities. In the sense of a stepped care model, low‐threshold treatment, for example, provided by nurses, could be a first step, and those who do not improve substantially should be allocated to more intensive treatment (Baglioni et al., 2023).

There is also a need for direct comparisons between brief treatment and full CBT‐I to determine non‐inferiority, for the full population of those with insomnia and subgroups. Hertenstein and colleagues are currently conducting a non‐inferiority trial comparing full CBT‐I with a shorter intervention that includes only bedtime restriction and stimulus control. Additional studies should focus on barriers to implementation, such as delivery modes (telehealth, digital, group versus individual), long‐term effects and relapse prevention. Another important area is predicting treatment response, such as identifying baseline factors like (subjective) sleep duration and mental health status that may inform tailored adjustments.

Addressing these questions will help clarify which populations benefit most from BBT‐I, where it can reduce costs and alleviate healthcare saturation, and which patients may need the full scope of CBT‐I.

## 5 RESEARCH AGENDA

**FIGURE 1:**
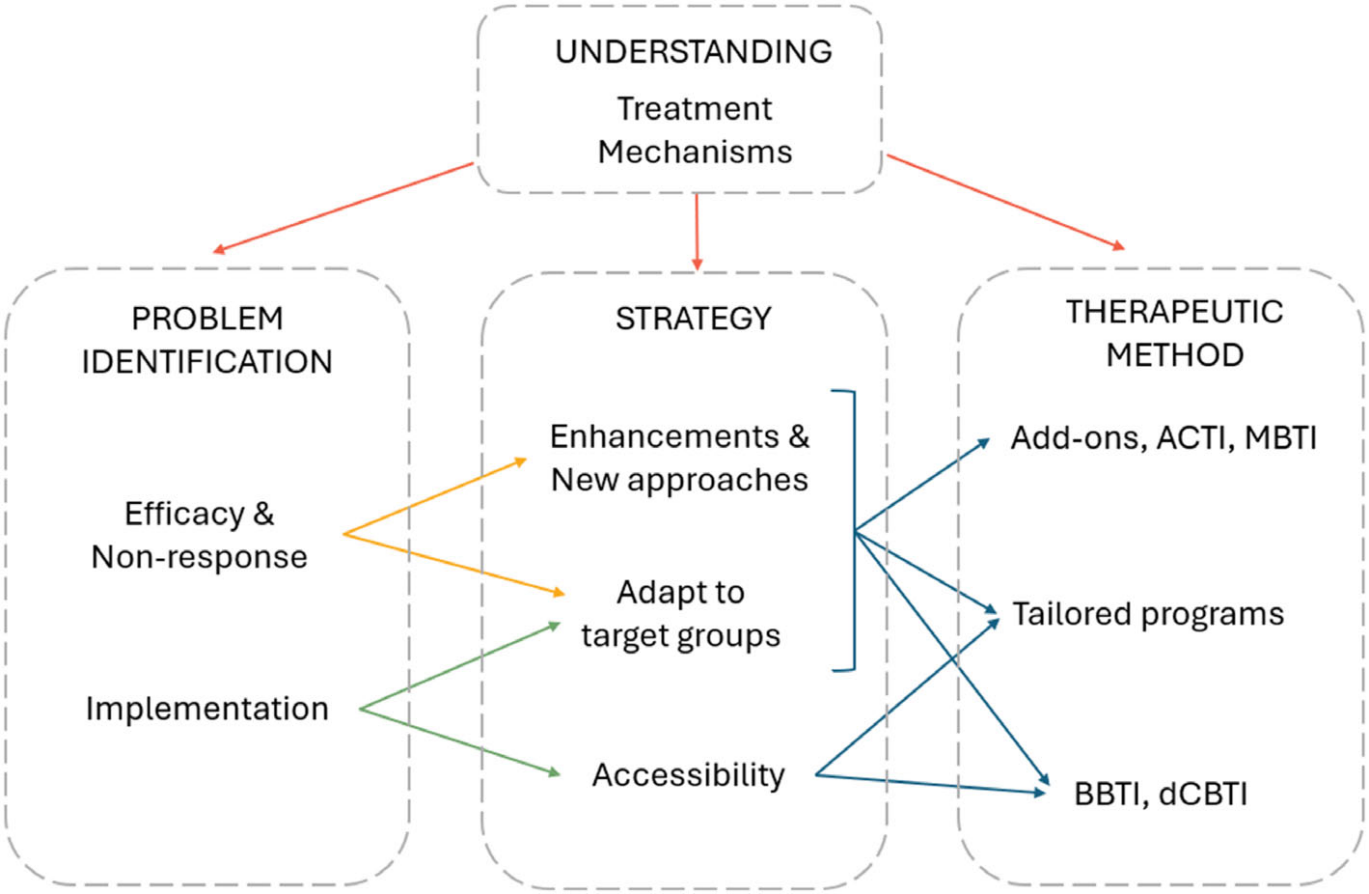
Overview of a research agenda for novel psychotherapies for insomnia. Integrated view of strategies and methods that may help overcome the main challenges at hand.

This article gives an extensive but non‐exhaustive overview of the current state of evidence of psychotherapeutic treatment of insomnia, including the current main challenges at hand. In our view, focusing on the aspects of enhancing/optimizing CBT‐I, addressing non‐response, and organizing widespread implementation will help to bring the field forward. In conclusion, we think it is important to make explicit once more, that for now CBT‐I is the undisputed first‐line treatment for patients with chronic insomnia—a treatment with good efficacy and tolerability that is mostly well accepted by patients as well as healthcare providers. We do, however, think that CBT‐I can be improved and that adjustments and/or alternative treatments may prove to enhance insomnia care in general. However, many of these additions first need further development and empirical testing. We would like to end this paper by summarizing the main practice points and a research agenda on key areas for future research and development with the aim of optimizing CBT‐I. Figure 1 gives an overview of the main challenges at hand, namely optimizing efficacy, addressing non‐response and improving implementation, and suggests strategies and methods that may help overcome these challenges (Table 1).

**TABLE 1:** Summary of the main points outlined in this article.

Summary of practice points
CBT‐I is the current treatment of first choice. Research into treatment mechanisms can help identify the most effective components and may inform about differential efficacy. Behavioural treatment alone and/or digital (C)BT‐I should be investigated as low‐cost alternative first‐line treatments. Adaptive treatment strategies can help tailor treatment according to individual needs.
Acceptance and commitment therapy (ACT) should be investigated as a potential second‐line treatment for those not responding to CBT‐I.
Exercise and circadian rhythm‐based strategies are potential add‐ons to CBT‐I that may help enhance efficacy.
Strategies from implementation science should be used to help identify barriers and facilitators to implementation. Brief treatment, digital treatment and tailored treatment may be easier to implement than face‐to‐face full CBT‐I, but further research is needed to fully understand factors impeding widespread implementation.

Abbreviations: ACT, acceptance and commitment therapy; CBT‐I, cognitive behavioural therapy for insomnia.

## AUTHOR CONTRIBUTIONS

**Marie Angelillo:** Writing – original draft; visualization. **Jaap Lancee:** Writing – review and editing; conceptualization. **Elisabeth Hertenstein:** Conceptualization; writing – original draft; writing – review and editing; supervision.

## Data Availability

Data sharing not applicable to this article as no datasets were generated or analysed during the current study.

## References

[jsr14470-bib-0001] American Psychiatric Association . (2013). Diagnostic and statistical manual of mental disorders (DSM‐5®). American Psychiatric Pub.

[jsr14470-bib-0002] Baglioni, C. , Espie, C. A. , Altena, E. , Gavriloff, D. , Jernelöv, S. , Holzinger, B. , Schlarb, A. , & Riemann, D. (2023). Cognitive behavioural therapy for insomnia disorder: Extending the stepped care model. Journal of Sleep Research, 32, e14016. 37584390 10.1111/jsr.14016

[jsr14470-bib-0003] Baron, K. G. , Abbott, S. , Jao, N. , Manalo, N. , & Mullen, R. (2017). Orthosomnia: Are some patients taking the quantified self too far? Journal of Clinical Sleep Medicine, 13, 351–354. 27855740 10.5664/jcsm.6472 PMC5263088

[jsr14470-bib-0004] Bensen‐Boakes, D.‐B. , Murali, T. , Lovato, N. , Lack, L. , & Scott, H. (2023). Wearable device‐delivered intensive sleep retraining as an adjunctive treatment to kickstart cognitive‐behavioral therapy for insomnia. Sleep Medicine Clinics, 18, 49–57. 36764786 10.1016/j.jsmc.2022.09.006

[jsr14470-bib-0005] Blanken, T. F. , Van Der Zweerde, T. , Van Straten, A. , Van Someren, E. J. , Borsboom, D. , & Lancee, J. (2019). Introducing network intervention analysis to investigate sequential, symptom‐specific treatment effects: a demonstration in co‐occurring insomnia and depression. Psychotherapy and Psychosomatics, 88(1), 52–54. 30625483 10.1159/000495045 PMC6469840

[jsr14470-bib-0006] Borbély, A. A. (1982). A two process model of sleep regulation. Human Neurobiology, 1, 195–204. 7185792

[jsr14470-bib-0007] Borbély, A. A. , Daan, S. , Wirz‐Justice, A. , & Deboer, T. (2016). The two‐process model of sleep regulation: A reappraisal. Journal of Sleep Research, 25, 131–143. 26762182 10.1111/jsr.12371

[jsr14470-bib-0008] Borghese, F. , Henckaerts, P. , Guy, F. , Perez Mayo, C. , Delplanque, S. , Schwartz, S. , & Perogamvros, L. (2022). Targeted memory reactivation during REM sleep in patients with social anxiety disorder. Frontiers in Psychiatry, 13, 904704. 35845468 10.3389/fpsyt.2022.904704 PMC9281560

[jsr14470-bib-0009] Bramoweth, A. D. , Lederer, L. G. , Youk, A. O. , Germain, A. , & Chinman, M. J. (2020). Brief behavioral treatment for insomnia vs. cognitive behavioral therapy for insomnia: Results of a randomized noninferiority clinical trial among veterans. Behavior Therapy, 51, 535–547. 32586428 10.1016/j.beth.2020.02.002 PMC10352919

[jsr14470-bib-0010] Callaway, C. A. , Sarfan, L. D. , Gumport, N. B. , & Harvey, A. G. (2023). The impact of module dosage on treatment response in a modular transdiagnostic intervention for sleep and circadian dysfunction (TranS‐C). Behaviour Research and Therapy, 168, 104368. 37478529 10.1016/j.brat.2023.104368

[jsr14470-bib-0011] Chambe, J. , Reynaud, E. , Maruani, J. , Fraih, E. , Geoffroy, P. A. , & Bourgin, P. (2023). Light therapy in insomnia disorder: A systematic review and meta‐analysis. Journal of Sleep Research, 32, e13895. 37002704 10.1111/jsr.13895

[jsr14470-bib-0012] Chiang, A. A. , & Khosla, S. (2023). Consumer wearable sleep trackers. Sleep Medicine Clinics, 18, 311–330. 37532372 10.1016/j.jsmc.2023.05.005

[jsr14470-bib-0013] Cienfuegos, S. , Gabel, K. , Kalam, F. , Ezpeleta, M. , Pavlou, V. , Lin, S. , Wiseman, E. , & Varady, K. A. (2022). The effect of 4‐h versus 6‐h time restricted feeding on sleep quality, duration, insomnia severity and obstructive sleep apnea in adults with obesity. Nutrition and Health, 28, 5–11. 33759620 10.1177/02601060211002347 PMC8460695

[jsr14470-bib-0014] Edinger, J. D. , Arnedt, J. T. , Bertisch, S. M. , Carney, C. E. , Harrington, J. J. , Lichstein, K. L. , Sateia, M. J. , Troxel, W. M. , Zhou, E. S. , Kazmi, U. , Heald, J. L. , & Martin, J. L. (2021). Behavioral and psychological treatments for chronic insomnia disorder in adults: An American Academy of sleep medicine clinical practice guideline. Journal of Clinical Sleep Medicine, 17, 255–262. 33164742 10.5664/jcsm.8986 PMC7853203

[jsr14470-bib-0015] Edinger, J. D. , Wamboldt, F. S. , Johnson, R. L. , Simmons, B. , Tsai, S. , Morin, C. M. , & Holm, K. E. (2023). Adherence to behavioral recommendations of cognitive behavioral therapy for insomnia predicts medication use after a structured medication taper. Journal of Clinical Sleep Medicine, 19, 1495–1503. 37086054 10.5664/jcsm.10616 PMC10394369

[jsr14470-bib-0016] Edinger, J. D. , Wohlgemuth, W. K. , Radtke, R. A. , Coffman, C. J. , & Carney, C. E. (2007). Dose‐response effects of cognitive‐behavioral insomnia therapy: A randomized clinical trial. Sleep, 30, 203–212. 17326546 10.1093/sleep/30.2.203

[jsr14470-bib-0017] El Rafihi‐Ferreira, R. , Hasan, R. , Toscanini, A. C. , Linares, I. M. P. , Suzuki Borges, D. , Brasil, I. P. , Carmo, M. , Lotufo Neto, F. , & Morin, C. (2024). Acceptance and commitment therapy versus cognitive behavioral therapy for insomnia: A randomized controlled trial. Journal of Consulting and Clinical Psychology, 92, 330–343. 39023982 10.1037/ccp0000881

[jsr14470-bib-0018] Ell, J. , Schmid, S. R. , Benz, F. , & Spille, L. (2023). Complementary and alternative treatments for insomnia disorder: A systematic umbrella review. Journal of Sleep Research, 32, e13979. 37527850 10.1111/jsr.13979

[jsr14470-bib-0019] Espie, C. A. , Broomfield, N. M. , MacMahon, K. M. A. , Macphee, L. M. , & Taylor, L. M. (2006). The attention‐intention‐effort pathway in the development of psychophysiologic insomnia: A theoretical review. Sleep Medicine Reviews, 10, 215–245. 16809056 10.1016/j.smrv.2006.03.002

[jsr14470-bib-0020] Espie, C. A. , Emsley, R. , Kyle, S. D. , Gordon, C. , Drake, C. L. , Siriwardena, A. N. , Cape, J. , Ong, J. C. , Sheaves, B. , Foster, R. , Freeman, D. , Costa‐Font, J. , Marsden, A. , & Luik, A. I. (2019). Effect of digital cognitive behavioral therapy for insomnia on health, psychological well‐being, and sleep‐related quality of life: A randomized clinical trial. JAMA Psychiatry, 76, 21–30. 30264137 10.1001/jamapsychiatry.2018.2745 PMC6583463

[jsr14470-bib-0021] Espie, C. A. , & Henry, A. L. (2023). Disseminating cognitive behavioural therapy (CBT) for insomnia at scale: Capitalising on the potential of digital CBT to deliver clinical guideline care. Journal of Sleep Research, 32, e14025. 37642008 10.1111/jsr.14025

[jsr14470-bib-0022] Forsell, E. , Jernelöv, S. , Blom, K. , Kraepelien, M. , Svanborg, C. , Andersson, G. , Lindefors, N. , & Kaldo, V. (2019). Proof of concept for an adaptive treatment strategy to prevent failures in internet‐delivered CBT: A single‐blind randomized clinical trial with insomnia patients. The American Journal of Psychiatry, 176, 315–323. 30696270 10.1176/appi.ajp.2018.18060699

[jsr14470-bib-0023] Furukawa, Y. , Sakata, M. , Yamamoto, R. , Nakajima, S. , Kikuchi, S. , Inoue, M. , Ito, M. , Noma, H. , Takashina, H. N. , Funada, S. , Ostinelli, E. G. , Furukawa, T. A. , Efthimiou, O. , & Perlis, M. (2024). Components and delivery formats of cognitive behavioral therapy for chronic insomnia in adults: A systematic review and component network meta‐analysis. JAMA Psychiatry, 81, 357–365. 38231522 10.1001/jamapsychiatry.2023.5060 PMC10794978

[jsr14470-bib-0024] Gunn, H. E. , Tutek, J. , & Buysse, D. J. (2019). Brief behavioral treatment of insomnia. Sleep Medicine Clinics, 14, 235–243. 31029189 10.1016/j.jsmc.2019.02.003

[jsr14470-bib-0025] Haghayegh, S. , Khoshnevis, S. , Smolensky, M. H. , Diller, K. R. , & Castriotta, R. J. (2019). Accuracy of wristband Fitbit models in assessing sleep: Systematic review and meta‐analysis. Journal of Medical Internet Research, 21, e16273. 31778122 10.2196/16273 PMC6908975

[jsr14470-bib-0026] Harvey, A. G. , Bélanger, L. , Talbot, L. , Eidelman, P. , Beaulieu‐Bonneau, S. , Fortier‐Brochu, É. , Ivers, H. , Lamy, M. , Hein, K. , Soehner, A. M. , Mérette, C. , & Morin, C. M. (2014). Comparative efficacy of behavior therapy, cognitive therapy, and cognitive behavior therapy for chronic insomnia: A randomized controlled trial. Journal of Consulting and Clinical Psychology, 82, 670–683. 24865869 10.1037/a0036606 PMC4185428

[jsr14470-bib-0027] Hasan, F. , Tu, Y.‐K. , Yang, C.‐M. , James Gordon, C. , Wu, D. , Lee, H. C. , Yuliana, L. T. , Herawati, L. , Chen, T. J. , & Chiu, H. Y. (2022). Comparative efficacy of digital cognitive behavioral therapy for insomnia: A systematic review and network meta‐analysis. Sleep Medicine Reviews, 61, 101567. 34902820 10.1016/j.smrv.2021.101567

[jsr14470-bib-0028] Hertenstein, E. , Thiel, N. , Lüking, M. , Külz, A. K. , Schramm, E. , Baglioni, C. , Spiegelhalder, K. , Riemann, D. , & Nissen, C. (2014). Quality of life improvements after acceptance and commitment therapy in nonresponders to cognitive behavioral therapy for primary insomnia. Psychotherapy and Psychosomatics, 83, 371–373. 25323449 10.1159/000365173

[jsr14470-bib-0072] Hertenstein, E. , Trinca, E. , Schneider, C. L. , Fehér, K. D. , Johann, A. F. , & Nissen, C. (2024). Acceptance and commitment therapy, combined with bedtime restriction, versus cognitive behavioral therapy for Insomnia: A randomized controlled pilot trial. Psychotherapy & Psychosomatics, 93, 114–128. 38417415 10.1159/000535834

[jsr14470-bib-0071] Jansson‐Fröjmark, M. , & Norell‐Carke, A. (2018). The cognitive treatment components and therapies of cognitive behavioral therapy for insomnia: A systematic review. Sleep Medicine Reviews, 42, 19–36. 29887256 10.1016/j.smrv.2018.05.001

[jsr14470-bib-0029] Jansson‐Frojmark, M. , Nordenstam, L. , Alfonsson, S. , Bohman, B. , Rozental, A. , & Norell‐Clarke, A. (2023). Stimulus control for insomnia: A systematic review and meta‐analysis. Journal of Sleep Research, 33(1), e14002. 37496454 10.1111/jsr.14002

[jsr14470-bib-0030] Krone, L. B. , Fehér, K. D. , Rivero, T. , & Omlin, X. (2023). Brain stimulation techniques as novel treatment options for insomnia: A systematic review. Journal of Sleep Research, 32, e13927. 37202368 10.1111/jsr.13927 PMC10909439

[jsr14470-bib-0031] Kyle, S. D. , Siriwardena, A. N. , Espie, C. A. , Yang, Y. , Petrou, S. , Ogburn, E. , Begum, N. , Maurer, L. F. , Robinson, B. , Gardner, C. , Lee, V. , Armstrong, S. , Pattinson, J. , Mort, S. , Temple, E. , Harris, V. , Yu, L. M. , Bower, P. , & Aveyard, P. (2023). Clinical and cost‐effectiveness of nurse‐delivered sleep restriction therapy for insomnia in primary care (HABIT): A pragmatic, superiority, open‐label, randomised controlled trial. Lancet, 402, 975–987. 37573859 10.1016/S0140-6736(23)00683-9

[jsr14470-bib-0032] Lack, L. , Scott, H. , & Lovato, N. (2019). Intensive sleep retraining treatment of insomnia. Sleep Medicine Clinics, 14, 245–252. 31029190 10.1016/j.jsmc.2019.01.005

[jsr14470-bib-0033] Lack, L. C. , Micic, G. , & Lovato, N. (2023). Circadian aspects in the aetiology and pathophysiology of insomnia. Journal of Sleep Research, 32, e13976. 37537965 10.1111/jsr.13976

[jsr14470-bib-0034] Lai, M. Y. C. , Mong, M. S. A. , Cheng, L. J. , & Lau, Y. (2023). The effect of wearable‐delivered sleep interventions on sleep outcomes among adults: A systematic review and meta‐analysis of randomized controlled trials. Nursing & Health Sciences, 25, 44–62. 36572659 10.1111/nhs.13011

[jsr14470-bib-0035] Lancee, J. , Harvey, A. G. , Morin, C. M. , Ivers, H. , van der Zweerde, T. , & Blanken, T. F. (2022). Network intervention analyses of cognitive therapy and behavior therapy for insomnia: Symptom specific effects and process measures. Behaviour Research and Therapy, 153, 104100. 35462241 10.1016/j.brat.2022.104100

[jsr14470-bib-0036] Lancee, J. , Morin, C. M. , Edinger, J. D. , Ivers, H. , van der Zweerde, T. , & Blanken, T. F. (2023). Symptom‐specific effects of zolpidem and behavioral treatment for insomnia: A network intervention analysis. Sleep, 46, zsad240. 37691423 10.1093/sleep/zsad240 PMC10636246

[jsr14470-bib-0037] Leerssen, J. , Lakbila‐Kamal, O. , Dekkers, L. M. S. , Ikelaar, S. L. C. , Albers, A. C. W. , Blanken, T. F. , Lancee, J. , van der Lande, G. J. M. , Maksimovic, T. , Mastenbroek, S. E. , Reesen, J. E. , van de Ven, S. , van der Zweerde, T. , Foster‐Dingley, J. C. , & van Someren, E. J. W. (2022). Treating insomnia with high risk of depression using therapist‐guided digital cognitive, behavioral, and circadian rhythm support interventions to prevent worsening of depressive symptoms: A randomized controlled trial. Psychotherapy and Psychosomatics, 91, 168–179. 34872087 10.1159/000520282

[jsr14470-bib-0038] Looman, M. I. , Blanken, T. F. , Schoenmakers, T. M. , et al. (n.d.). Telephone‐guided sleep restriction for insomnia: A randomized sleep diary‐controlled trial. submitted manuscript. 10.1159/000545138 PMC12060811 40122034

[jsr14470-bib-0039] Looman, M. I. , Schoenmakers, T. M. , Blanken, T. F. , Linnebank, F. E. , Kamphuis, J. H. , & Lancee, J. (2024). Efficacy of acceptance and commitment therapy as a stand‐alone treatment for insomnia: Protocol of a randomized waitlist controlled trial. Journal of Behavioral and Cognitive Therapy, 34, 100499.

[jsr14470-bib-0040] Luik, A. I. , Farias Machado, P. , & Espie, C. A. (2018). Delivering digital cognitive behavioral therapy for insomnia at scale: Does using a wearable device to estimate sleep influence therapy? npj Digital Medicine, 1, 1–6. 31304289 10.1038/s41746-017-0010-4 PMC6548338

[jsr14470-bib-0041] Luik, A. I. , van der Zweerde, T. , van Straten, A. , & Lancee, J. (2019). Digital delivery of cognitive behavioral therapy for insomnia. Current Psychiatry Reports, 21, 50. 31161406 10.1007/s11920-019-1041-0 PMC6546653

[jsr14470-bib-0042] Maurer, L. F. , Schneider, J. , Miller, C. B. , Espie, C. A. , & Kyle, S. D. (2021). The clinical effects of sleep restriction therapy for insomnia: A meta‐analysis of randomised controlled trials. Sleep Medicine Reviews, 58, 101493. 33984745 10.1016/j.smrv.2021.101493

[jsr14470-bib-0043] Meng, J. , Xiao, X. , Wang, W. , Jiang, Y. , Jin, Y. , & Wang, H. (2023). Sleep quality, social rhythms, and depression among people living with HIV: A path analysis based on social zeitgeber theory. Frontiers in Psychiatry, 14, 14. 10.3389/fpsyt.2023.1102946 PMC10192574 37215662

[jsr14470-bib-0044] Monk, T. H. , Reynolds, I. I. I. , Charles, F. , Buysse, D. J. , DeGrazia, J. M. , & Kupfer, D. J. (2003). The relationship between lifestyle regularity and subjective sleep quality. Chronobiology International, 20, 97–107. 12638693 10.1081/cbi-120017812

[jsr14470-bib-0045] Morin, C. M. , Belleville, G. , Bélanger, L. , & Ivers, H. (2011). The insomnia severity index: Psychometric indicators to detect insomnia cases and evaluate treatment response. Sleep, 34, 601–608. 21532953 10.1093/sleep/34.5.601 PMC3079939

[jsr14470-bib-0046] Morin, C. M. , Edinger, J. D. , Beaulieu‐Bonneau, S. , Ivers, H. , Krystal, A. D. , Guay, B. , Bélanger, L. , Cartwright, A. , Simmons, B. , Lamy, M. , & Busby, M. (2020). Effectiveness of sequential psychological and medication therapies for insomnia disorder: A randomized clinical trial. JAMA Psychiatry, 77, 1107. 32639561 10.1001/jamapsychiatry.2020.1767 PMC7344835

[jsr14470-bib-0047] Morin, C. M. , Vallières, A. , Guay, B. , Ivers, H. , Savard, J. , Mérette, C. , Bastien, C. , & Baillargeon, L. (2009). Cognitive behavioral therapy, singly and combined with medication, for persistent insomnia: A randomized controlled trial. Journal of the American Medical Association, 301, 2005–2015. 19454639 10.1001/jama.2009.682 PMC3050624

[jsr14470-bib-0048] Mulder, F. , Löwinger, D. , Jenkinson, S. P. , Kaiser, E. , Scharf, T. , Maire, M. , Duss, S. , Bassetti, C. , Heinzer, R. , Auer, R. , & Meyer‐Massetti, C. (2023). Counselling for chronic insomnia in Swiss pharmacies: A survey study based on case vignettes. Pharmacy (Basel), 11, 105. 37368431 10.3390/pharmacy11030105 PMC10302654

[jsr14470-bib-0049] Nunes, M. E. B. , dos Santos, C. H. B. , de Oliveira Lima, M. , Pedrosa, A. K. P. , de Menezes, R. C. E. , & Longo‐Silva, G. (2024). Association of Evening Eating with sleep quality and insomnia among adults in a Brazilian National Survey. Sleep Science, 17(4), e381–e391. 39698174 10.1055/s-0044-1779605 PMC13155930

[jsr14470-bib-0050] Ong, J. C. , & Kalmbach, D. A. (2023). Mindfulness as an adjunct or alternative to CBT‐I. Sleep Medicine Clinics, 18, 59–71. 36764787 10.1016/j.jsmc.2022.09.002

[jsr14470-bib-0051] Ong, J. C. , Manber, R. , Segal, Z. , Xia, Y. , Shapiro, S. , & Wyatt, J. K. (2014). A randomized controlled trial of mindfulness meditation for chronic insomnia. Sleep, 37, 1553–1563. 25142566 10.5665/sleep.4010 PMC4153063

[jsr14470-bib-0052] Ong, J. C. , Ulmer, C. S. , & Manber, R. (2012). Improving sleep with mindfulness and acceptance: A metacognitive model of insomnia. Behaviour Research and Therapy, 50, 651–660. 22975073 10.1016/j.brat.2012.08.001 PMC3466342

[jsr14470-bib-0053] Ong, J. C. , Xia, Y. , Smith‐Mason, C. E. , & Manber, R. (2018). A randomized controlled trial of mindfulness meditation for chronic insomnia: Effects on daytime symptoms and cognitive‐emotional arousal. Mindfulness, 9, 1702–1712.

[jsr14470-bib-0054] Passos, G. S. , Youngstedt, S. D. , & Santana, M. G. (2023). Exercise as an adjunct treatment to cognitive behavior therapy for insomnia. Sleep Medicine Clinics, 18, 39–47. 36764785 10.1016/j.jsmc.2022.09.001

[jsr14470-bib-0055] Paulos‐Guarnieri, L. , Linares, I. M. P. , & El Rafihi‐Ferreira, R. (2022). Evidence and characteristics of acceptance and commitment therapy (ACT)‐based interventions for insomnia: A systematic review of randomized and non‐randomized trials. Journal of Contextual Behavioral Science, 23, 1–14.

[jsr14470-bib-0056] Perini, F. , Wong, K. F. , Lin, J. , Hassirim, Z. , Ong, J. L. , Lo, J. , Ong, J. C. , Doshi, K. , & Lim, J. (2023). Mindfulness‐based therapy for insomnia for older adults with sleep difficulties: A randomized clinical trial. Psychological Medicine, 53, 1038–1048. 34193328 10.1017/S0033291721002476 PMC9975962

[jsr14470-bib-0057] Riedel, A. , Benz, F. , Deibert, P. , Barsch, F. , Frase, L. , Johann, A. F. , Riemann, D. , & Feige, B. (2024). The effect of physical exercise interventions on insomnia: A systematic review and meta‐analysis. Sleep Medicine Reviews, 76, 101948. 38749363 10.1016/j.smrv.2024.101948

[jsr14470-bib-0058] Riemann, D. , Espie, C. A. , Altena, E. , Arnardottir, E. S. , Baglioni, C. , Bassetti, C. L. A. , Bastien, C. , Berzina, N. , Bjorvatn, B. , Dikeos, D. , Dolenc Groselj, L. , Ellis, J. G. , Garcia‐Borreguero, D. , Geoffroy, P. A. , Gjerstad, M. , Gonçalves, M. , Hertenstein, E. , Hoedlmoser, K. , Hion, T. , … Spiegelhalder, K. (2023). The European insomnia guideline: An update on the diagnosis and treatment of insomnia 2023. Journal of Sleep Research, 32, e14035. 38016484 10.1111/jsr.14035

[jsr14470-bib-0059] Schneider, C. L. , Hertenstein, E. , Fehér, K. , Maier, J. G. , Cantisani, A. , Moggi, F. , Berger, T. , & Nissen, C. (2020). *Become your own SLEEPexpert*: Design, implementation, and preliminary evaluation of a pragmatic behavioral treatment program for insomnia in inpatient psychiatric care. Sleep Advances, 1, zpaa005. 37192879 10.1093/sleepadvances/zpaa005 PMC10104352

[jsr14470-bib-0060] Schwartz, S. , Clerget, A. , & Perogamvros, L. (2022). Enhancing imagery rehearsal therapy for nightmares with targeted memory reactivation. Current Biology, 32, 4808–4816.e4. 36306786 10.1016/j.cub.2022.09.032

[jsr14470-bib-0061] Scott, H. , Cheung, J. M. Y. , Muench, A. , Ivers, H. , Grandner, M. A. , Morin, C. M. , & Perlis, M. L. (2023). Baseline sleep characteristics are associated with gains in sleep duration after cognitive behavioral therapy for insomnia. Sleep Medicine, 102, 199–204. 36701834 10.1016/j.sleep.2023.01.009

[jsr14470-bib-0062] Scott, H. , Lechat, B. , Manners, J. , Lovato, N. , Vakulin, A. , Catcheside, P. , Eckert, D. J. , & Reynolds, A. C. (2023). Emerging applications of objective sleep assessments towards the improved management of insomnia. Sleep Medicine, 101, 138–145. 36379084 10.1016/j.sleep.2022.10.030

[jsr14470-bib-0063] Sheaves, B. , Freeman, D. , Isham, L. , McInerney, J. , Nickless, A. , Yu, L. M. , Rek, S. , Bradley, J. , Reeve, S. , Attard, C. , Espie, C. A. , Foster, R. , Wirz‐Justice, A. , Chadwick, E. , & Barrera, A. (2018). Stabilising sleep for patients admitted at acute crisis to a psychiatric hospital (OWLS): An assessor‐blind pilot randomised controlled trial. Psychological Medicine, 48, 1694–1704. 29108526 10.1017/S0033291717003191 PMC6088775

[jsr14470-bib-0064] Steinmetz, L. , Simon, L. , Feige, B. , Riemann, D. , Johann, A. F. , Ell, J. , Ebert, D. D. , Baumeister, H. , Benz, F. , & Spiegelhalder, K. (2024). Network meta‐analysis examining efficacy of components of cognitive behavioural therapy for insomnia. Clinical Psychology Review, 114, 102507. 39504928 10.1016/j.cpr.2024.102507

[jsr14470-bib-0065] Swanson, L. M. , & Raglan, G. B. (2023). Circadian interventions as adjunctive therapies to cognitive‐behavioral therapy for insomnia. Sleep Medicine Clinics, 18, 21–30. 36764783 10.1016/j.jsmc.2022.09.004 PMC10015491

[jsr14470-bib-0066] Troxel, W. M. , Conrad, T. S. , Germain, A. , & Buysse, D. J. (2013). Predictors of treatment response to brief behavioral treatment of insomnia (BBTI) in older adults. Journal of Clinical Sleep Medicine, 9, 1281–1289. 24340290 10.5664/jcsm.3270 PMC3836339

[jsr14470-bib-0067] Tyagi, S. , Resnick, N. M. , Perera, S. , Monk, T. H. , Hall, M. H. , & Buysse, D. J. (2014). Behavioral treatment of chronic insomnia in older adults: Does nocturia matter? Sleep, 37, 681–687. 24899759 10.5665/sleep.3568 PMC4044748

[jsr14470-bib-0068] van Egmond, L. T. , Moulin, T. C. , Schiöth, H. B. , Cederholm, T. , & Benedict, C. (2020). Meal timing and subjective sleep disturbances in older men. Experimental Gerontology, 141, 111089. 32911034 10.1016/j.exger.2020.111089

[jsr14470-bib-0069] van Straten, A. , van der Zweerde, T. , Kleiboer, A. , Cuijpers, P. , Morin, C. M. , & Lancee, J. (2018). Cognitive and behavioral therapies in the treatment of insomnia: A meta‐analysis. Sleep Medicine Reviews, 38, 3–16. 28392168 10.1016/j.smrv.2017.02.001

[jsr14470-bib-0070] Yan, L.‐M. , Li, H.‐J. , Fan, Q. , Xue, Y.‐D. , & Wang, T. (2024). Chronobiological perspectives: Association between meal timing and sleep quality. PLoS One, 19, e0308172. 39088487 10.1371/journal.pone.0308172 PMC11293727

